# The index of severity for eosinophilic esophagitis reflects treatment response in children and associates with outcome variables

**DOI:** 10.1007/s00431-025-06159-9

**Published:** 2025-05-05

**Authors:** Carolina Gutiérrez-Junquera, Alejandro García-Díaz, Sonia Fernández-Fernández, Guadalupe Tena-García, Angela Marazuela, Elisabet Díez-Vela, Maria Luz Cilleruelo, Enriqueta Román

**Affiliations:** 1 Pediatric Gastroenterology Unit, Hospital Universitario Puerta de Hierro-Majadahonda, Calle Manuel de Falla 1, 28224 Majadahonda, Madrid, Spain; 2 Universidad Autónoma de Madrid, Madrid, Spain; 3 Pediatric Gastroenterology Unit. Hospital Universitario Severo Ochoa, Leganés, Madrid, Spain

**Keywords:** Eosinophilic esophagitis, Severity, I-SEE, Treatment, Outcomes

## Abstract

We aimed to categorize the severity of eosinophilic esophagitis using the recently developed Index of Severity of Eosinophilic Esophagitis (I-SEE), to assess the longitudinal response to treatment, and to correlate baseline severity with outcome variables. A retrospective analysis of a prospectively enrolled cohort of children at two centers was performed. I-SEE was calculated at diagnosis, at the second endoscopy after initial treatment, and at the last endoscopy over a mean of 35 months. We analyzed clinical, endoscopic, and histologic characteristics at baseline by disease severity, examined the change in severity at the second and last endoscopy, and evaluated the association of baseline disease severity with treatment variables. Of 95 children meeting inclusion criteria, 35%, 63%, and 2% had mild, moderate, and severe I-SEE scores, respectively, at baseline. Between baseline and the second endoscopy, the median I-SEE decreased from 7 (7–8) to 2 (0–5) (*p* < 0.001), and to 0 (0–3) at last endoscopy, with 53% of patients having inactive and 47% having mild scores (*p* < 0.001). Patients with histologic response at the second and final endoscopy had lower I-SEE scores than non-responders (*p* < 0.001). A higher baseline I-SEE score was associated with increased odds of receiving 2 or more treatments or combined therapy (OR 95% CI 1.28 (1.03–1.59) and (1.18 (1.04–1.39), respectively).

*Conclusions*: The I-SEE score reflects longitudinal treatment response in children with EoE. Patients with higher baseline I-SEE may be at higher risk of requiring more than one treatment, sequentially or in combination.

**Table:** 

**What is Known**: • *Eosinophilic esophagitis is a heterogenous disease with different degrees of severity.* • *The recently developed index of severity of eosinophilic esophagitis (I-SEE) incorporates frequency of symptoms, complications, and inflammatory and fibrostenotic findings and has shown to be a sensitive tool to change with treatment in a pediatric cohort.*	
**What is New**: • *We confirmed the sensitivity of the index, with a significant decrease in the score with treatment, with the decrease being more pronounced in children with histologic response.* • *A higher initial severity score was associated with the need for more treatments, sequentially or in combination.*

**Supplementary Information:**

The online version contains supplementary material available at 10.1007/s00431-025-06159-9.

## Introduction

Eosinophilic esophagitis (EoE) is an emerging chronic disease, characterized by symptoms of esophageal dysfunction and esophageal mucosal inflammation with a predominant eosinophilic infiltrate [1–3]. EoE is a heterogeneous entity, with variations in symptoms, endoscopic, and histologic findings among patients, that may show a predominant inflammatory or fibrostenotic phenotype. Furthermore, response to treatment also varies, with some cases of refractory disease.

Validated scoring systems have been developed to categorize symptoms [4], endoscopic [5], and histologic findings [6]. Recently, a clinical severity index (I-SEE) has been proposed for patients with EoE, taking into account the frequency of symptoms, the presence of complications, and the presence of inflammatory or fibrostenotic findings at the endoscopic and histologic levels [7, 8].

To date, the clinical applicability of this index has been studied in adults in the short term [9], and in a cohort of children followed in the longer term [10]. A recent study found a correlation between the I-SEE and molecular features of EoE, suggesting clinical relevance of the index [11]. More data on the clinical applicability of the I-SEE in real-life cohorts of patients are needed, especially in the pediatric age group, as there are important differences in the clinical characteristics of EoE between children and adults [12].

The objectives of this study are to categorize the severity of EoE at diagnosis using the I-SEE in a cohort of pediatric patients at diagnosis, to assess the longitudinal response of the I-SEE to treatment, and to correlate the baseline severity with outcome variables.

## Materials and methods

### Patient selection

We performed a retrospective cohort study of pediatric EoE patients prospectively enrolled in the longitudinal database of two hospitals: Hospital Universitario Puerta de Hierro Majadahonda and Hospital Universitario Severo Ochoa, Madrid, Spain. Patients were diagnosed between January 2017 and March 2022. Study data were collected in the REDCap electronic data capture tools (Vanderbilt University) [13] hosted at Sociedad Española de Gastroenterología, Hepatología y Nutrición Pediátrica (redcap.seghnp.org) with the support of the AEGREDCap Support Unit and shared with the Asociación Española de Gastroenterología.

Inclusion criteria were the following: (1) age of 1–18 years, a new diagnosis of EoE according to consensus diagnostic criteria [symptoms of esophageal dysfunction and eosinophilic infiltration of esophageal mucosa > 15 eosinophils (eos)/high power field (hpf)] and other causes of eosinophilia excluded [2], (2) at least three esophagogastroduodenoscopies (EGD) performed (at diagnosis, after initial treatment and on maintenance therapy), and (3) follow-up of at least 1 year. No exclusion criteria were applied.

### Treatment protocol

Patients at both centers were treated according to the same protocol based on international and national recommendations [1, 14]. First-line monotherapy with either proton pump inhibitors (PPIs), an elimination diet, or swallowed topical corticosteroids (STCs) was initiated, according to each patient’s characteristics in a shared decision-making process. The standard management protocol included a follow-up endoscopy 3–4 months after treatment initiation. The decision to change treatment was based on symptoms, endoscopic findings, and histologic data. Combined treatment (PPIs plus STCs, or PPIs plus diet) was used after failure of first-line monotherapy with PPI, STCs and/or diet in a shared decision-making process with patients and families. A follow-up endoscopy was usually performed 3–6 months after each treatment change. In patients who achieved clinical, endoscopic and histologic response to treatment, endoscopic surveillance was typically performed every 1–3 years. Histologic response was defined as the presence of < 15 eosinophils/hpf (HPF area 0.3 mm^2^).

### Clinical data and I-SEE calculation

The calculation of the I-SEE was performed for each domain using data included in REDCap and data extracted from medical records. The frequency of symptoms and the presence of complications (e.g., food impaction, malnutrition, EoE-related hospitalization, esophageal perforation, and treatment-refractory disease) were documented from the time of endoscopy before any change in treatment. Endoscopic inflammatory features (localized or diffuse edema, furrows, and exudates) and fibrostenotic features (strictures, rings, and dilation) were derived from the EoE Endoscopic Reference Score (EREFS) recorded in the endoscopic notes. Histologic inflammatory and fibrostenotic scores were calculated based on data obtained from pathology reports. In the RedCap database eosinophilic infiltration was registered in four categories: < 15; 15–50; 51–100 and > 100 eos/hpf.

I-SEE scores were calculated at baseline diagnostic endoscopy (I-SEE 1), at the time of the second endoscopy after initial treatment (I-SEE 2), and at the last endoscopic performed (I-SEE 3). Patients were categorized as inactive (0 points), mild (1–6 points), moderate (7–14 points), or severe (≥ 15–78 points) [7].

Demographic parameters, patient and family history, baseline clinical symptoms, endoscopic and histologic findings, initial treatment, number of treatments received, administration of combined treatment, and number of endoscopies performed per year, were also recorded.

### Statistical analysis

Descriptive analysis was performed using medians and interquartile ranges [IQRs] for numerical variables and absolute and relative frequencies for categorical variables. Univariate analysis was performed using the Kruskal–Wallis test when the categorical variable had more than two categories and the Mann–Whitney *U* test when there were two categories. Wilcoxon nonparametric paired ranks tests were also performed. Multivariable analysis was performed by logistic regression, with effect size expressed as odds ratio (OR) and the corresponding 95% confidence interval (95%CI). The evolution of I-SEE over time was also evaluated using a generalizing estimating equations (GEE) model, reflecting the evolution of the I-SEE score at each time point with respect to diagnosis, adjusting for peak eosinophil count. Linear regression analysis was performed to evaluate the association between the I-SEE score at a given time and diagnosis. The significance level was set at *p* < 0.05. Stata/IC version 18 software (Stata Corp LLC, College Station, TX, USA) was used for the analyses.

## Results

Between January 2017 and March 2022, 128 children were diagnosed with EoE, of which 95 met the inclusion criteria and had complete data available to generate the I-SEE score; 24% were female, median age was 11 (8.1–14) years.

### Baseline patients characteristics and eosinophilic esophagitis disease severity

At baseline, the median I-SEE score (I-SEE 1) was 7 (6–8), corresponding to a category of moderate severity. Thirty-three patients (35%) were in the mild category, 60 (63%) in the moderate category, and only 2 (2%) in the severe category (Table 1). Regarding complications, 16 patients (17%) presented with food impaction requiring an emergency department visit (Table 2). No patient presented with malnutrition (body mass index < 5 th percentile or decreased growth trajectory), and the 2 patients classified as severe presented with esophageal perforation. One patient presented with thoracic pain with suspected small perforation on chest CT, which resolved with conservative management. In the other patient, esophageal dissection was observed at the passage of the endoscope which was closed with endoscopic clips. Regarding endoscopic findings, 91 (96%) patients showed a diffuse inflammatory endoscopic pattern, with only 9 patients (9%) showing a fibrostenotic phenotype. However, 67 patients (70%) had fibrostenotic histologic findings, mostly due to the presence of basal zone hyperplasia (Table 2).

**Table 1:** Baseline clinical features by severity, as classified by I-SEE at diagnosis (I-SEE 1)

	Mild	Moderate	Severe	Total	*p* value
Patients	33 (35)	60 (63)	2 (2)	95 (100)	
I-SEE score Median (IQR)	5 (5–6)	8 (7–9)	23 (20–26)	7 (6–8)	< 0.001
Age Median (IQR)	11 (8.6–13)	12 (7–14)	11 (10–13)	11 (8.1–14)	0.87
Female *n* (%)	6 (18)	16 (27)	1 (50)	23 (24)	0.34
Body mass index (BMI) Percentile Median (IQR)	50 (21–71)	55 (35–84)	20 (15–25)	53 (26–77)	0.15
Symptoms onset time *n* (%)					
< 6 months	11 (33)	14 (23)	0 (0)	25 (26)	0.64
6–12 months	5 (15)	15 (25)	0 (0)	20 (21)	
> 12 months	16 (48)	30 (50)	2 (100)	48 (50)	
Any atopic background *n* (%)	18 (54)	41 (68)	1 (50)	60 (63)	0.32
Food allergy *n* (%)	7 (21)	14 (23)	1 (50)	22 (23)	0.59
Symptoms *n* (%)					
Pyrosis	15 (45)	25 (42)	1 (50)	41 (43)	0.91
Abdominal pain	14 (42)	25 (42)	1 (50)	40 (42)	1.0
Vomiting/nausea	14 (42)	17 (28)	1 (50)	32 (34)	0.26
Poor feeding	10 (30)	17 (28)	2 (100)	29 (30)	0.16
Dysphagia	17 (51)	36 (60)	2 (100)	55 (58)	0.48
Food impaction	14 (42)	31 (52)	2 (100)	47 (49)	0.29
Weight loss	2 (6)	6 (10)	1 (50)	9 (9)	0.18
Retrosternal pain	3 (9)	10 (17)	1(50)	14 (15)	0.20
Endoscopic findings *n* (%)					
Normal endoscopy	3 (9)	1 (2)	0 (0)	4 (4)	0.19
Edema	29 (88)	53 (88)	1 (50)	83 (87)	0.32
Exudates	21 (64)	50 (83)	2 (100)	73 (77)	0.09
Furrows	30 (91)	58 (97)	2 (100)	90 (95)	0.41
Rings	0 (0)	6 (10)	0 (0)	6 (6)	0.19
Stenosis	0 (0)	3 (5)	1 (50)	4 (4)	0.06
Histologic findings *n* (%)					
Peak eosinophil count 15–50 51–100 > 100	20 (61) 10 (30) 3 (9)	18 (30) 24 (40) 18 (30)	0 (0) 2 (100) 0 (0)	38 (40) 36 (38) 21 (22)	0.007
Initial treatment n (%)					0.08
PPIs	31 (94)	50 (83)	1 (50)		
Elimination diet	2 (6)	6 (10)	0 (0)		
STCs	0 (0)	4 (6)	1 (50)		

*I-SEE*, Index of severity of Eosinophilic Esophagitis; *IQR*, Interquartile range; *BMI*, body mass index; *PPIs*, proton pump inhibitors; *STCs*, swallowed topical steroids

**Table 2:** Comparison of severity index at baseline (I-SEE 1), after initial treatment (I-SEE 2) and at last endoscopy (I-SEE 3)

	Baseline I-SEE 1	After initial treatment I-SEE 2	*p* value^a^	Last endoscopy I-SEE 3	*p* value^b^
Time, mean SD (months)		3.44 ± 1.1		35.3 ± 15.0	
I-SEE, median (IQR)	7 (6–8)	2 (0–5)	< 0.001	0 (0–3)	< 0.001
Category, *n* (%) Inactive Mild Moderate Severe	0 (0) 33 (35) 60 (63) 2 (2)	33 (35) 51 (54) 11 (11) 0 (0)	< 0.001	50 (53) 45 (47) 0 0	< 0.001
Symptoms, *n* (%) None Weekly Daily Multiple times per day/disruptive	1 (1) 56 (59) 31 (33) 7 (7)	74 (78) 15 (16) 6 (6) 0 (0)	< 0.001	85 (90) 8 (8) 2 (2) 0 (0)	< 0.001
Complications, *n* (%) Food impaction with ED visit/endoscopy Hospitalization Perforation Malnutrition Persistent inflammation requiring elemental formula, systemic steroids or immunomodulatory treatments	16 (17) 0 (0) 2 (2) 0 (0) 0 (0)	0 (0) 0 (0) 0 (0) 0 (0) 0 (0)	< 0.001	1 (10) 0 (0) 0 (0) 0 (0) 0 (0)	< 0.001
Inflammatory features, *n* (%)					
Endoscopy (edema, furrows, exudates)			< 0.001		< 0.001
None	4 (4)	40 (42)		64 (67)	
Localized	7 (7)	21 (22)		17 (18)	
Diffuse	84 (89)	34 (36)		14 (15)	
Histology, *n* (%)			< 0.001		19 (20)
< 15	0 (0)	51 (54)		69 (73)	
15–60	43 (45)	25 (26)		17 (18)	
> 60	52 (55)	19 (20)		9 (9)	
Fibrostenotic features, *n* (%)					
Endoscopy (rings, strictures)			0.0191		0.0332
None	86 (91)	93 (98		92 (97)	
Present but scopes passes easily	5 (5)	1 (1)		3 (3)	
Present, but requires dilation or a snug fit when passing a standard endoscope	4 (4)	1 (1)		0 (0)	
Cannot pass standard endoscope or repeated dilations (adult) or any dilation (child)	0 (0)	0 (0)		0 (0)	
Histology, *n* (%)			< 0.001		< 0.001
(BZH or LPF; DEC/SEA if no LP)	67 (70)	36 (38)		25 (26)	

*ER*, emergency department; *I-SEE*, Index of severity of Eosinophilic Esophagitis; *IQR*, Interquartile range; *BZH*, basal zone hyperplasia; *LPF*, lamina propria fibrosis; *DEC*, dyskeratotic cells; *SEA*, surface epithelial alteration; *LP*, lamina propria

a: ISEE 2 vs I-SEE1

b: I-SEE 3 vs I-SEE1

We observed no differences in baseline I-SEE categories with respect to age, sex, time of symptom onset, family or personal history of atopy, or food allergy, frequency of each symptom, BMI percentile or inflammatory endoscopic findings (Table 1). The presence of rings was detected in only 6 moderate cases; and stenosis in 3 moderate cases and 1 severe case, compared with none in the mild category.

Only 13 patients (39%) of the mild cases had eosinophil quantification (peak value) > 50 eos/hpf at diagnosis, compared to 42 (70%) of the moderate cases (*p* = 0.007).

Regarding initial treatment, 82 patients (81%) received PPIs at a dose of 1 mg/kg/day BID. None of the patients classified as mild received STCs at diagnosis, compared to 4% of those classified as moderate (*p* = 0.007).

### Longitudinal evolution of I-SEE

#### After initial treatment (I-SEE 2)

**Fig. 1:**
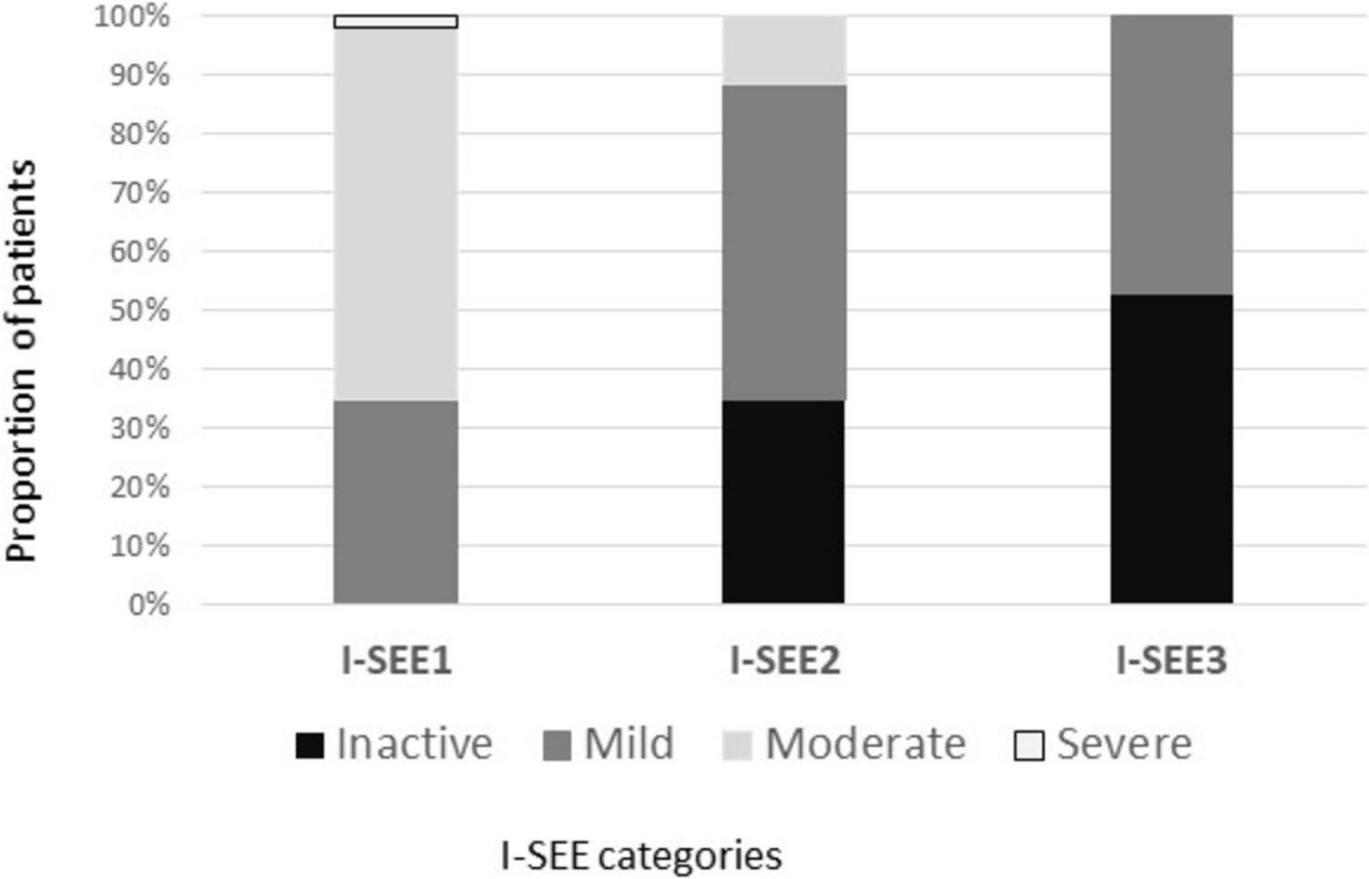
Percentage of patients in each I-SEE category at baseline (I-SEE 1); after initial treatment (I-SEE2) and last endoscopy (I-SEE 3)

After initial treatment and a follow-up time of 3.4 ± 1.1 months, I-SEE decreased from 7 (6–8) to 2 (0–5), (*p* < 0.001). The I-SEE severity classification also improved with 35%, 54%, 11%, and 0% showing inactive, mild, moderate and severe disease, respectively, compared to 0%, 35%, 63%, and 2% at baseline endoscopy (*p* < 0.001) (Table 2, Fig. 1).

We observed a significant decrease in all I-SEE domains after treatment in both symptom frequency (*p* < 0.001), complications (*p* < 0.001), endoscopic inflammatory and histologic findings (*p* < 0.001); and endoscopic fibrostenotic findings (*p* = 0.02) (Table 2). There was also a significant decrease in the percentage of patients with histologic fibrostenosis after initial treatment (70% vs. 38%, *p* < 0.001). No patient developed complications during this period.

#### At last follow-up endoscopy (I-SEE 3)

At the third endoscopy, after a mean follow-up time of 35.3 ± 15.0 months, the I-SEE score decreased significantly from baseline: 7 (6–8) to 0 (0–3) (*p* < 0.001) with 53% of patients classified as inactive and the remaining 47% as mild (*p* < 0.001) (Table 2, Fig. 1).

A significant decrease was also observed in all I-SEE domains from baseline endoscopy to the last endoscopy, both in the frequency of symptoms (*p* < 0.001), complications (*p* < 0.001), inflammatory findings both endoscopic and histologic (*p* < 0.001); and endoscopic fibrostenotic findings (*p* = 0.03) (Table 2). There was a significant decrease in the percentage of patients with histologic fibrostenosis findings from baseline I-SEE after treatment (70% vs. 26%, *p* < 0.001).

One patient experienced a complication consisting of food impaction and emergency department visit with spontaneous resolution without endoscopy.

### Association of I-SEE2 and I-SEE3 with histologic response

We observed a significantly lower value of I-SEE 2 and I-SEE 3 in those patients with histologic response (< 15 eos/hpf) compared to those without histologic response (Table 3), even considering that lack of histologic response would score a maximum of 2 points. Similarly, all patients with histologic response were classified as inactive or mildly active at both initial and final follow-up (*p* < 0.001, Table 3).

**Table 3:** Severity category assessed by I-SEE after induction treatment (I-SEE 2) and at last endoscopy (I-SEE 3) according to histologic response

	Histologic responders	Histologic nonresponders	*p* value
After induction treatment (ISEE 2)	N = 50	N = 45	
Median (IQR)	0 (0–1)	5 (3–6)	< 0.001
Category			< 0.001
Inactive	33 (66)	0 (0)	
Mild	17 (34)	34 (76)	
Moderate	0 (0)	11 (24)	
Severe	0 (0)	0 (0)	
At last endoscopy (I-SEE 3)	N = 70	N = 25	
Median (IQR)	0 (0–1)	5 (3–5)	< 0.001
Category			< 0.001
Inactive	50 (71)	0	
Mild	20 (29)	25 (100)	
Moderate	0 (0)	0 (0)	
Severe	0 (0)	0 (0)	

*I-SEE*, Index of severity of Eosinophilic Esophagitis; *IQR*, Interquartile range

### Association of baseline I-SEE with follow-up variables

Patients with mild category at diagnosis received a median of 1 treatment, versus moderate or severe who received 2 treatments per patient (*p* < 0.004) (Table 4). However, we did not observe differences in the number of endoscopies per year in the three categories at diagnosis. Only one patient (3%) of patients with mild baseline I-SEE received combined treatment, compared to 15 (25%) of those classified as moderate and 1 (50%) of those classified as severe (*p* < 0.006). Combined treatment consisted of PPIs plus STCs in 10 patients and elimination diet plus PPIs in the remaining seven patients. Sixty-seven percent of patients classified as mild at diagnosis achieved histologic response with initial treatment compared to 43% of moderate patients (p = 0.039). However, we observed no association between the initial I-SEE category and histologic response at the last follow-up endoscopy.

**Table 4:** Outcome of patients classified by baseline I-SEE (I-SEE 1)

	Mild (*n* = 33)	Moderate (*n* = 60)	Severe (*n* = 2)	*p*
Number of treatments (median, interquartile range)	1 (1–2)	2 (1–3)	2 (2–2)	0.004
Combined therapy (*n*, %)	1 (3)	15 (25)	1 (50)	0.006
Number of endoscopies/year (median, interquartile range)	(1.5–2.5)	2.1 (1.7–2.5)	1.8 (1.5–2.2)	0.43
< 15 eos/hpf after induction treatment	22 (67)	26 (43)	2 (100)	0.039
< 15 eos/hpf at last endoscopy	25 (76)	43 (71)	2 (100)	0.63

*I-SEE*, Index of severity of Eosinophilic Esophagitis; *IQR*, Interquartile range

In the multivariable analysis, we observed that, after adjustment for sex, age and personal history of atopy, the variables number of treatments received and combined treatment were statistically significantly associated with I-SEE at diagnosis (I-SEE 1). Thus, for each additional point of I-SEE 1, both the odds of receiving 2 or more treatments and of receiving combined therapy increased (OR 95% CI 1.28 (1.03–1.59) and (1.18 (1.04–1.39), respectively. However, no association was observed with histologic response after initial treatment.

On the contrary, we did not observe an association with peak eosinophils/hpf at diagnosis and number of treatments required, number of endoscopies per year, administration of combined therapy, or histologic response after initial treatment (Supplementary Table).

### Prediction analysis

**Fig. 2:**
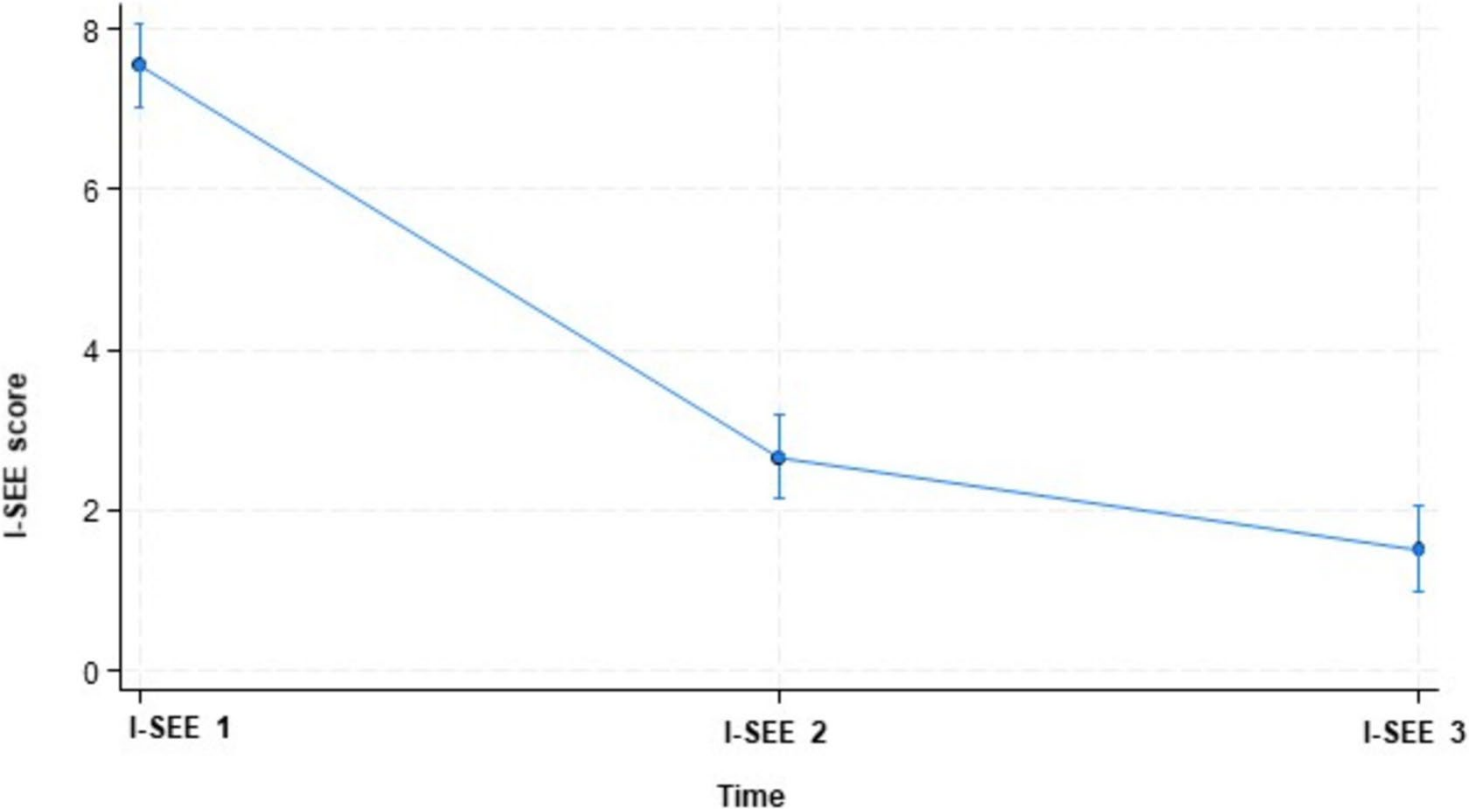
Evolution of I-SEE after initial treatment (I-SEE 2) and at the last endoscopy (I-SEE3), adjusting for peak eosinophils/hpf at diagnosis

In the longitudinal analysis, the evolution of I-SEE was predicted after initial treatment (I-SEE 2) and at the last endoscopy (I-SEE 3), adjusting for peak eosinophils/hpf at diagnosis. Compared to I-SEE 1, a decrease in I-SEE 2 of − 4.88 (95%CI, − 5–61 to − 4.15, *p* < 0.001) and I-SEE 3 of − 6.03 (95%CI − 6.76 to − 5.30, *p* < 0.001) was estimated (Fig. 2).

Linear regression analysis did not show an association between a higher baseline I-SEE score (I-SEE 1) and higher subsequent scores after induction treatment (I-SEE 2, *β* = 0.55 for a 1-point change in I-SEE1; 95% confidence interval [CI] − 0. 12 to 0.23, *p* = 0.54), or higher scores at last endoscopy (I-SEE 3, (I-SEE2, *β* = − 0.39; 95% confidence interval [CI] − 0.17 to 0.094, *p* = 0.56), after adjustment for maximum peak of eosinophils/hpf in baseline biopsies.

## Discussion

The I-SEE severity index was recently developed to categorize the severity of EoE [7]. The present study is the first to evaluate the applicability of the I-SEE severity index in a population of European children newly diagnosed with EoE. Compared to previous research, it includes the largest pediatric cohort to date, and its multicenter design may imply a more diverse patient population. In our longitudinally followed cohort of children, we have observed that the I-SEE behaved as a treatment-sensitive tool, with a significant decrease after treatment. More interestingly, the presence of a higher baseline I-SEE was associated with several prognostic variables, such as the need for more than one treatment sequentially or in combination.

In our study, most of the children (63%) had moderate EoE at diagnosis, and only 2% had severe disease. These data contrast with those published in the previous study conducted in the USA in which 21% of the patients were classified as severe at diagnosis. In this study, greater severity was associated with the presence of malnutrition in 15% of the cases, that correlated with the presence of poor feeding and low BMI percentile [10]. In contrast, we did not observe malnutrition at diagnosis, probably because the children in our cohort were older than those in the US cohort (median 11 years vs. mean 5.2 years). It is known that the faltering growth associated with EoE is more common in younger children [15, 16]. Two patients with severe EoE in our study presented with esophageal perforation, a rare complication in children [17].

Although we observed that only 39% of mild cases presented with eosinophil quantification > 50 eos/hpf at diagnosis, compared to 70% of those classified as moderate, we did not have the exact number of eos/hpf to compare between groups. In the two previous published studies, no significant differences in baseline peak eosinophil counts/hpf were observed between the various categories at diagnosis, suggesting that there is no correlation between baseline eosinophil count and severity of EoE [9, 10].

We observed that the I-SEE is a responsive tool, with a decrease in the index globally and in all domains, reflecting a response to treatment. The decrease in I-SEE was progressive over time, with all patients being classified as mild or inactive at the last endoscopy after a mean follow-up of 35 months. Our results confirmed that the decrease in the severity index with treatment was greater in patients with histologic response; both after initial treatment and at the last follow-up, although eosinophilic inflammation contributed only to a maximum of 2 points to the I-SEE score [9, 10].

Although there is a decrease in all domains of the I-SEE, we have observed that the percentage of patients with positive scores in the histologic fibrostenosis domain remained high both after the first treatment (38%) and in the last follow-up (26%). These data contrast with 2 and 3% of patients with positive scores for fibrostenotic endoscopic features, respectively. In most cases, the histologic fibrostenotic finding score is achieved by the presence of basal zone hyperplasia. The presence of basal zone hyperplasia, along with dyskeratotic epithelial cells and surface epithelial alterations, may predict lamina propria fibrosis in cases where lamina propria is absent [18, 19]. Furthermore, it may contribute to long-term remodeling in biopsies from children with EoE [20] and may associate with clinical and endoscopic data in patients without eosinophilic infiltration after treatment [21]. The observed discordance between endoscopic and histologic data regarding fibrostenosis may indicate that a longer treatment period may be required for resolution of basal cell hyperplasia or lamina propria fibrosis. However, it also raises the question of whether it would be necessary to separate the data of lamina propria fibrosis and the presence of basal cell hyperplasia in I-SEE. On the other hand, a grading of basal cell hyperplasia could be established from which it would be considered significant.

Interestingly, our data suggests that baseline I-SEE may correlate with certain prognostic variables. Thus, a higher baseline I-SEE correlated with a higher frequency of administration of combined treatment and a greater number of treatments required during follow-up. On the contrary, the peak eosinophil count at diagnosis was not associated with these variables, emphasizing that a higher peak eosinophil count is not associated with greater severity of EoE. In addition, our data suggests that the severity of baseline I-SEE may influence the choice of the initial treatment, with STCs administered only to children with moderate or severe baseline I-SEE. This supports the usefulness of the score, since pediatric gastroenterologists in Europe generally indicate STCs as a second treatment, except in more severe patients, with severe dysphagia or fibrostenotic phenotype [22] [23].

The main limitations of this study are its retrospective nature, which may limit the data collected, such as the frequency of symptoms, and the unavailability of the exact figure quantifying eos/hpf, since it was registered as categories in the database. Another limitation is the lack of data on treatment adherence, as these data were collected from routine clinical practice and not from a clinical trial. However, the strengths of the study include the treatment and follow-up of patients with a common protocol, of monotherapy with one of the therapeutic modalities and agreed on drug doses and follow-up time, with change of treatment in case of no response and if this persisted, administration of combined treatment. Moreover, the sample represents 75% of the patients diagnosed and followed up in the participating hospitals during this period.

In conclusion, our results support the usefulness of the I-SEE to assess the severity of EoE in children at baseline, and to monitor the response to different therapeutic interventions in routine clinical practice. Patients with higher baseline severity index may be at higher risk of requiring more than one treatment sequentially or in combination. Further studies are needed to prospectively assess the applicability of the I-SEE in children to guide therapy, the need to implement the different domains, and the value of the I-SEE to support changes in treatment.

## Supplementary Information

Below is the link to the electronic supplementary material.

ESM 1 (DOCX 15.8 KB)

## Data Availability

Data is provided within the manuscript.

## Abbreviations

*BZH*: Basal zone hyperplasia
*BMI*: Body mass index
*CI*: Confidence interval
*DEC*: Dyskeratotic cells
*EGD*: Esophagogastroduodenoscopy
*EoE*: Eosinophilic esophagitis
*Eos*: Eosinophils
*EREFS*: EoE endoscopic reference score
*IQR*: Interquartile ranges
*I-SEE*: Index of severity of eosinophilic esophagitis
*hpf*: High power field
*LP*: Lamina propria
*LPF*: Lamina propria fibrosis
*PPIs*: Proton pump inhibitors
*STCs*: Swallowed topical corticosteroids
*OR*: Odds ratio
*SEA*: Surface epithelial alteration
