# Supplementary material for: The index of severity for eosinophilic esophagitis reflects treatment response in children and associates with outcome variables
Source: Eur J Pediatr. 2025 May 5;184(6):325. doi: 10.1007/s00431-025-06159-9 (PMC12053194; doi:10.1007/s00431-025-06159-9)
Supplement: Supplementary file 1 — (DOCX 15.8 KB) [file 431_2025_6159_MOESM1_ESM.docx]

Supplementary table. Outcome of patients classified by baseline peak eosinophil count

|  | 15-50  n=38 | 51-100  n=36 | >100  n=21 | p value |
| --- | --- | --- | --- | --- |
| Number of treatments  Median (IQR) | 1.9 (1.4-2.2) | 2.2 (1.5-2.5) | 2.2 (1.8-2.6) | 0.37 |
| Combined therapy, n, (%) | 9 (24) | 6 (17) | 2 (9.5) | 0.39 |
| Number of endoscopies/year  Median (IQR) | 2 (1-2) | 2 (1-2.5) | 2 (1.3) | 0.23 |
| < 15 eos/hpf after induction treatment, n (%) | 22 (58) | 19 (33) | 9 (43) | 0.54 |
| < 15 eos/hpf at last endoscopy, n (%) | 7 (18.4) | 15 (41) | 3(14) | 0.03 |

IQR: Interquartile range. eos: eosinophils. hpf: high power field.
